# Mitochondria and Cancer

**DOI:** 10.3390/cancers12092641

**Published:** 2020-09-16

**Authors:** Alexandr V. Bazhin

**Affiliations:** 1 Department of General, Visceral and Transplantation Surgery, Ludwig-Maximilians University, 81377 Munich, Germany; alexandr.bazhin@med.uni-muenchen.de; Tel.: +49-894-4007-3440; 2 German Cancer Consortium (DKTK), Partner Site Munich, 80336 Munich, Germany

Mitochondria are indispensable for energy metabolism and are essential for the regulation of many cellular processes in healthy as well as in transformed cells. Mitochondria in malignant cells differ structurally and functionally from those in normal cells, which make them a promising target for anticancer therapy. Mitochondria in cancer cells are characterized by reactive oxygen species (ROS) overproduction, which promotes cancer development by inducing genomic instability, modifying gene expression and participating in signaling pathways. Additionally, the pleiotropic roles of mitochondrial ROS in the regulation of anticancer immunity are now coming to light. The involvement of mitochondria in cancer cell metabolic reprogramming as well as in the anticancer immune response has been utilized for designing novel mitochondria-targeted anticancer agents. However, we are still far from an in depth understanding of the role played by mitochondria in cancer development. Therefore, the main aim of this Special Issue was to collect novel findings and ideas from scientists involved in basic research as well as in translational studies in the field of mitochondria and cancer. 

Hguen and Pandey [1] discussed profoundly in their review “Expoiting Mitochondrial Vulnerability to trigger Apoptosis Selectively in Cancer Cells” regarding recent mitochondrial-selective anticancer compounds with efficient toxicity against cancer cells. These compounds should create a vicious cycle of mitohondrial dysfunction leading to the production of reactive oxygen species (ROS) and finally to cell suicide. Furthermore, the authors conceptualized a possibility of the combination of these compounds for therapeutic usage.

Another interesting point in gender-related anticancer treatment is the topic of the review of Stakisaitis et al.: “The Importance of Gender-Related Anticancer Research on Mitochondrial regulator Sodium Dichloroacetate in Preclinical Studies In Vivo” [2]. The review describes an excellent example of such gender-related effect by cancer treatment with sodium dichloroacetate. The authors conclude that gender-related differences in pharmacology of drugs targeting cancer mitochondrion should be recognized especially in the context of an individualized therapy. 

Jeena et al. [3] hypothesized in their paper “Recent Progress in Mitochondria-Targeted Drug and Drug-Free Agents for Cancer Therapy” that certain structural differences in mitochondria between normal and cancer cells could be used for the designing of selective anticancer drugs. In respect of this assumption, the authors give us an overview about alternative drug-free approaches to target cancer mitochondria. The discussed approaches may open new avenues for unconventional strategies to combat cancer.

Autophagy is a key node for the regulation of ROS levels as well as for the ROS-dependent cellular regulation. Therefore, such regulation could be a potential target mechanism for cancer treatment. Based on this idea, Sanches-Ávarez et al. [4] recognize the sestrin family of proteins as a “missing link” between ROS and autophagy in cancer cells. In their review “Sestrins as a Therapeutic bridge between ROS and Autophagy in Cancer”, the authors discuss possibilities for the adaptive regulation of ROS-induced autophagy by sestrins. Finally, they propose synergistic strategies using the pharmacological regulation of these proteins for personalized anticancer therapy. 

The drug resistance, without question, is a huge barrier in the struggle against cancer. As previously mentioned, it is important to know the structural differences between normal and cancer cells. Moscheni et al. [5] also contemplated in the same way and investigated in their work “3D Quantitative and Ultrastructural Analysis of Mitochondria in a Model of Doxorubicin Sensitive and Resistant Human Colon Carcinoma Cells” the differences in the mitochondrial morphology in drug-resistant cells against drug-sensitive ones using the soft X-ray cryo nanotomography. Indeed, they found certain differences in the cell strains. It is certain that these data will be helpful for designing new anticancer strategies.

Finally, Meng et al. [6] in their research article “Oncogenic K-ras Induces Mitochondrial OPA3 Expression to promote Energy metabolism in Pancreatic Cancer Cells” present evidence that OPA3 might be involved in cellular energy metabolism, and its upregulation has a link to K-ras mutations. Such upregulation finally led to the modulation of cancer cell proliferation and of epithelial–mesenchymal transition. These data are important considering that pancreatic cancer is one of the deadliest cancers in the world. 

Summarizing, a small but high quality spectrum of reviews and original papers from this issue provides an insight into new research directions linked to an extremely important topic “Mitochondria and Cancer”. I hope all readers of this Special Issue enjoy taking a closer look into a subject of this compendium. 
